# 2-[(*E*)-2-(4-Hy­droxy-3-meth­oxy­phen­yl)ethen­yl]-1-methylpyridinium 4-bromo­benzene­sulfonate monohydrate

**DOI:** 10.1107/S1600536813031917

**Published:** 2013-11-30

**Authors:** Suchada Chantrapromma, Nawong Boonnak, Boonwasana Jindawong, Hoong-Kun Fun

**Affiliations:** a Department of Chemistry, Faculty of Science, Prince of Songkla University, Hat-Yai, Songkhla 90112, Thailand; b Faculty of Traditional Thai Medicine, Prince of Songkla University, Hat-Yai, Songkhla 90112, Thailand; c X-ray Crystallography Unit, School of Physics, Universiti Sains Malaysia, 11800 USM, Penang, Malaysia

## Abstract

The title salt crystallized as the monohydrate C_15_H_16_NO_2_^+^·C_6_H_4_BrSO_3_^−^·H_2_O. The cation exists in an *E* conformation with respect to the ethynyl bond and is essentially planar, with a dihedral angle of 6.52 (14)° between the pyridinium and the benzene rings. The hy­droxy and meth­oxy substituents are coplanar with the benzene ring to which they are attached, with an r.m.s. deviation of 0.0116 (3) Å for the nine non-H atoms [C_meth­yl_—O—C—C torsion angle = −0.8 (4)°]. In the crystal, the cations and anions are stacked by π–π inter­actions, with centroid–centroid distances of 3.7818 (19) and 3.9004 (17) Å. The cations, anions and water mol­ecules are linked by O—H⋯O hydrogen bonds and weak C—H⋯O inter­actions, forming a three-dimensional network.

## Related literature

For applications of stilbene derivatives, see: Chanawanno *et al.* (2010 ▶); Frombaum *et al.* (2012 ▶); Hussain *et al.* (2009 ▶); Jindawong *et al.* (2005 ▶); Kobkeatthawin *et al.* (2009 ▶); Li *et al.* (2013 ▶); Ruanwas *et al.* (2010 ▶). For related structures, see, Chanawanno *et al.* (2009 ▶); Chantrapromma *et al.* (2013 ▶); Fun *et al.* (2011 ▶). For bond-length data, see: Allen *et al.* (1987 ▶) and for hydrogen-bond motifs, see: Bernstein *et al.* (1995 ▶). For the stability of the temperature controller used in the data collection, see: Cosier & Glazer, (1986 ▶).
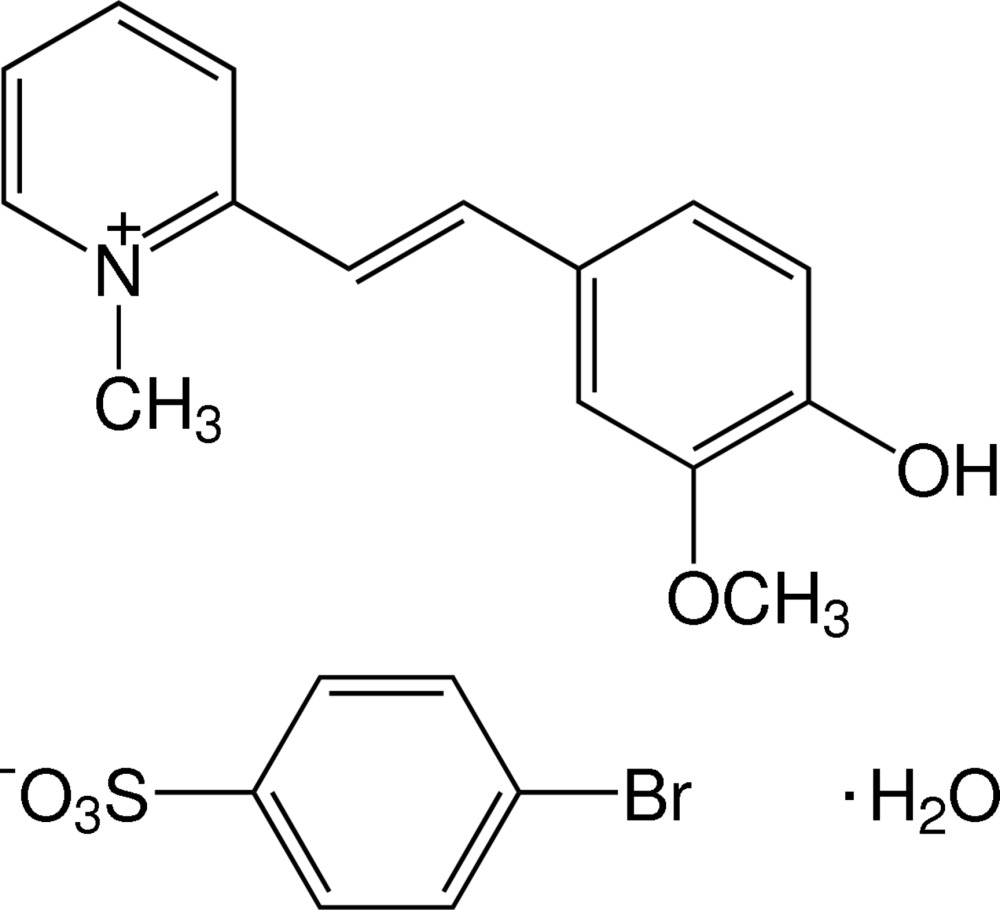


## Experimental

### 

#### Crystal data

C_15_H_16_NO_2_^+^·C_6_H_4_BrO_3_S^−^·H_2_O
*M* *_r_* = 496.37
Triclinic, 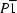

*a* = 9.8201 (13) Å
*b* = 10.3315 (14) Å
*c* = 12.4914 (17) Å
α = 99.898 (2)°
β = 111.134 (2)°
γ = 107.042 (2)°
*V* = 1074.1 (3) Å^3^
*Z* = 2
Mo *K*α radiation
μ = 2.05 mm^−1^
*T* = 100 K
0.59 × 0.15 × 0.14 mm

#### Data collection

Bruker APEXII CCD area-detector diffractometer
Absorption correction: multi-scan (*SADABS*; Bruker, 2005 ▶) *T*_min_ = 0.378, *T*_max_ = 0.768
11172 measured reflections
4186 independent reflections
3356 reflections with *I* > 2σ(*I*)
*R*_int_ = 0.021

#### Refinement

*R*[*F* ^2^ > 2σ(*F* ^2^)] = 0.044
*wR*(*F* ^2^) = 0.113
*S* = 1.05
4186 reflections
281 parameters
H atoms treated by a mixture of independent and constrained refinement
Δρ_max_ = 0.93 e Å^−3^
Δρ_min_ = −0.94 e Å^−3^

### 

Data collection: *APEX2* (Bruker, 2005 ▶); cell refinement: *SAINT* (Bruker, 2005 ▶); data reduction: *SAINT*; program(s) used to solve structure: *SHELXTL* (Sheldrick, 2008 ▶); program(s) used to refine structure: *SHELXTL*; molecular graphics: *SHELXTL*; software used to prepare material for publication: *SHELXTL*, *PLATON* (Spek, 2009 ▶), *Mercury* (Macrae *et al.*, 2006 ▶) and *publCIF* (Westrip, 2010 ▶).

## Supplementary Material

Crystal structure: contains datablock(s) global, I. DOI: 10.1107/S1600536813031917/sj5370sup1.cif

Structure factors: contains datablock(s) I. DOI: 10.1107/S1600536813031917/sj5370Isup2.hkl

Supplementary material file. DOI: 10.1107/S1600536813031917/sj5370Isup3.cml

Additional supplementary materials:  crystallographic information; 3D view; checkCIF report

## Figures and Tables

**Table 1:** Hydrogen-bond geometry (Å, °)

*D*—H⋯*A*	*D*—H	H⋯*A*	*D*⋯*A*	*D*—H⋯*A*
O2—H1*O*2⋯O1*W* ^i^	0.82	1.88	2.685 (4)	169
O1*W*—H2*W*1⋯O4^ii^	0.81 (5)	2.03 (5)	2.834 (4)	172 (5)
O1*W*—H1*W*1⋯O3^iii^	0.81 (4)	1.99 (5)	2.793 (5)	173 (5)
C1—H1*A*⋯O5^iv^	0.93	2.57	3.491 (4)	170
C2—H2*A*⋯O1^v^	0.93	2.51	3.440 (4)	176
C2—H2*A*⋯O2^v^	0.93	2.60	3.183 (4)	121
C3—H3*A*⋯O2^v^	0.93	2.54	3.160 (4)	124
C14—H14*A*⋯O4^iv^	0.96	2.54	3.448 (5)	158

Symmetry codes: (i) 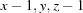; (ii) 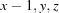; (iii) 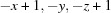; (iv) 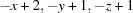; (v) 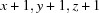.

**Fig. 1.**
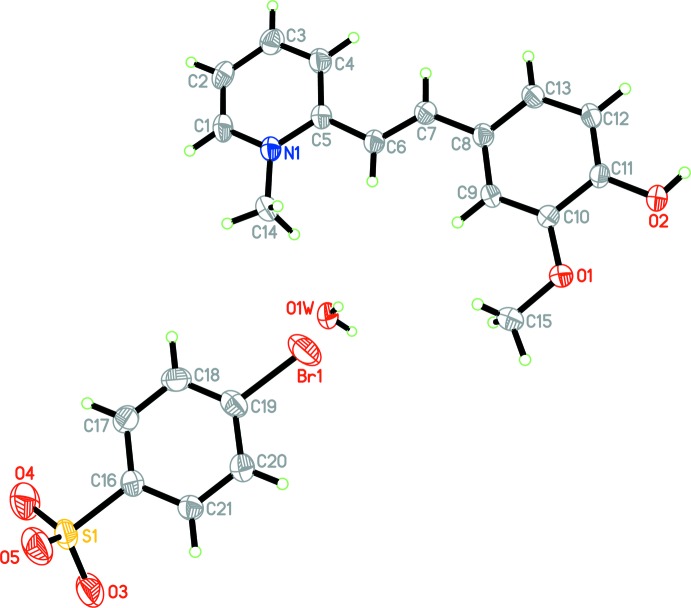
The molecular structure of the title compound, showing 30% probability displacement ellipsoids.

**Fig. 2.**
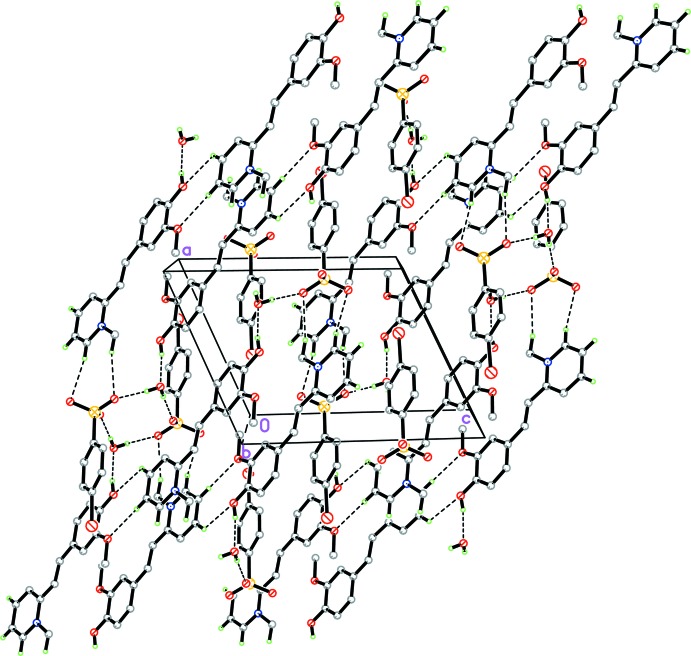
The crystal packing of the title compound viewed approximately along the b axis. Only H atoms involved in O—H···O hydrogen bonds and weak C—H···O interactions are shown for clarity. Hydrogen bonds are drawn as dashed lines.

**Fig. 3.**
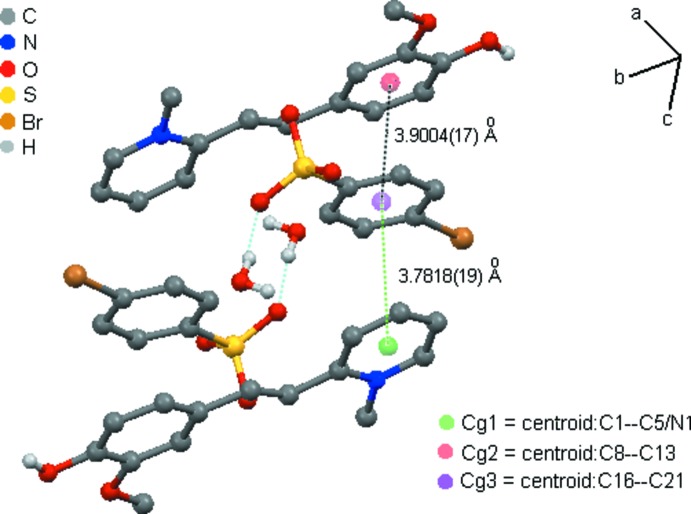
π–π interactions between aromatic rings of the cations and anions.

## supplementary crystallographic information

### 1. Comment

Stilbene-based compounds have been reported to possess a wide range of biological activities including antibacterial (Chanawanno *et al.*, 2010) and antioxidant (Frombaum *et al.*, 2012) activities and also non-linear optical (Ruanwas *et al.*, 2010) and fluorescent properties (Li *et al.*, 2013). We have previously reported several crystal structures and applications of stilbene derivatives (Chanawanno *et al.*, 2009; 2010, Kobkeatthawin *et al.*, 2009, Ruanwas *et al.*, 2010). Due to these interesting properties, the title pyridinium-stilbene salt, (I), was synthesized. We report herein the synthesis and crystal structure of (I).

The asymmetric unit of (I) consists of a C_15_H_16_NO_2_^+^ cation, a C_6_H_4_BrSO_3_^-^ anion and an H_2_O molecule (Fig. 1). The cation exists in an *E* configuration with respect to the C6 ═C7 double bond [1.318 (4) Å] and the C5—C6—C7—C8 torsion angle is -178.0 (3)°. The cation is essentially planar with a dihedral angle between the pyridinium and benzene rings of the cation being 6.52 (14)°. The hydroxy and methoxy substituents lie close to the plane of the C8–C13 benzene ring with the *r.m.s.* deviation of 0.0116 (3) Å for the nine non-H atoms and with the torsion angle C15–O1–C10–C9 = -0.8 (4)°. All bond lengths (Allen *et al.*, 1987) in both the cation and anion are normal and compare well with those found in closely related structures (Chanawanno *et al.*, 2009; Chantrapromma *et al.*, 2013; Fun *et al.*, 2011).

In the crystal packing (Fig. 2), weak C2—H2A···O1 and C3—H3A···O2 interactions (Table 1) link together two inversely-related adjacent cations, generating an *R*_2_^2^(8) ring motif (Bernstein *et al.*, 1995). The O1W—H1W1···O3 and O1W—H2W1···O4 hydrogen bonds (Table 1) linked between two water molecules and two anions forming an *R*_2_^4^(12) ring motif. The cations, anions and water molecules are further linked through intermolecular O–H···O hydrogen bonds and weak C—H···O interactions into a three dimensional network (Fig. 2 and Table 1). π–π interactions with distances *Cg*_1_···*Cg*_3_^vi^ = 3.7818 (19) Å and *Cg*_2_···*Cg*_3_^ii^ = 3.9004 (17) Å were observed (Fig. 3); *Cg*_1_, *Cg*_2_ and *Cg*_3_ are the centroids of C1–C5/N1, C8–C13 and C16–C21 rings, respectively [symmetry code (vi) = 1 - *x*, 1 - *y*, 1 - *z*].

### 2. Experimental

1-Methyl-2-[(*E*)-2-(3-methoxy-4-hydroxyphenyl)ethenyl]pyridinium iodide (compound A) was prepared by mixing a solution (1:1:1 molar ratio) of 1,2-dimethylpyridinium iodide (3.02 g, 12.84 mmol), vanillin (4-hydroxy-3-methoxybenzaldehyde, 1.95 g, 12.82 mmol) and piperidine (1.09 g, 12.80 mmol). The resulting solution was refluxed for 3 h under a nitrogen atmosphere. The solid which formed was filtered, washed with diethylether and recrystallized from methanol, to give brown crystals of compound A (2.69 g, 57% yield Mp. 526–527 K). Thereafter, the title compound was synthesized by mixing a solution of compound A (0.21 g, 0.58 mmol) in hot methanol (60 ml) and a solution of silver (I) 4-bromobenzenesulfonate (Jindawong *et al.*, 2005), (0.20 g, 0.58 mmol) in hot methanol (40 ml). Upon mixing, a yellow precipitate of silver iodide was immediately formed which was removed by filtration and the orange filtrate was evaporated under reduced pressure to yield the title compound as an orange solid (0.27 g, 91% yield). Orange block-shaped single crystals of the title compound suitable for X-ray structure determination were recrystallized from methanol by slow evaporation of the solvent at room temperature over several days, Mp. 490–491 K.

### 3. Refinement

Water H atoms were located in difference maps and refined isotropically. The remaining H atoms were positioned geometrically and allowed to ride on their parent atoms, with d(O—H) = 0.82 Å, d(C—H) = 0.93 Å for aromatic and CH, and 0.96 Å for CH_3_ atoms. The *U*_iso_ values were constrained to be 1.5*U*_eq_ of the carrier atom for methyl H atoms and 1.2*U*_eq_ for the remaining H atoms. A rotating group model was used for the methyl groups.

### Crystal data

**Table:**

C_15_H_16_NO_2_^+^·C_6_H_4_BrO_3_S^−^·H_2_O	*Z* = 2
*M**_r_* = 496.37	*F*(000) = 508
Triclinic, *P*1	*D*_x_ = 1.535 Mg m^−^^3^
Hall symbol: -P 1	Melting point = 490–491 K
*a* = 9.8201 (13) Å	Mo *K*α radiation, λ = 0.71073 Å
*b* = 10.3315 (14) Å	Cell parameters from 4186 reflections
*c* = 12.4914 (17) Å	θ = 1.8–26.0°
α = 99.898 (2)°	µ = 2.05 mm^−^^1^
β = 111.134 (2)°	*T* = 100 K
γ = 107.042 (2)°	Block, orange
*V* = 1074.1 (3) Å^3^	0.59 × 0.15 × 0.14 mm

### Data collection

**Table:**

Bruker APEXII CCD area-detector diffractometer	4186 independent reflections
Radiation source: sealed tube	3356 reflections with *I* > 2σ(*I*)
Graphite monochromator	*R*_int_ = 0.021
φ and ω scans	θ_max_ = 26.0°, θ_min_ = 1.8°
Absorption correction: multi-scan (*SADABS*; Bruker, 2005)	*h* = −12→12
*T*_min_ = 0.378, *T*_max_ = 0.768	*k* = −12→12
11172 measured reflections	*l* = −15→15

### Refinement

**Table:**

Refinement on *F*^2^	Primary atom site location: structure-invariant direct methods
Least-squares matrix: full	Secondary atom site location: difference Fourier map
*R*[*F*^2^ > 2σ(*F*^2^)] = 0.044	Hydrogen site location: inferred from neighbouring sites
*wR*(*F*^2^) = 0.113	H atoms treated by a mixture of independent and constrained refinement
*S* = 1.05	*w* = 1/[σ^2^(*F*_o_^2^) + (0.0432*P*)^2^ + 0.8763*P*] where *P* = (*F*_o_^2^ + 2*F*_c_^2^)/3
4186 reflections	(Δ/σ)_max_ = 0.001
281 parameters	Δρ_max_ = 0.93 e Å^−^^3^
0 restraints	Δρ_min_ = −0.94 e Å^−^^3^

### Special details

**Table:**

Experimental. The crystal was placed in the cold stream of an Oxford Cryosystems Cobra open-flow nitrogen cryostat (Cosier & Glazer, 1986) operating at 100.0 (1) K.
Geometry. All e.s.d.'s (except the e.s.d. in the dihedral angle between two l.s. planes) are estimated using the full covariance matrix. The cell e.s.d.'s are taken into account individually in the estimation of e.s.d.'s in distances, angles and torsion angles; correlations between e.s.d.'s in cell parameters are only used when they are defined by crystal symmetry. An approximate (isotropic) treatment of cell e.s.d.'s is used for estimating e.s.d.'s involving l.s. planes.
Refinement. Refinement of *F*^2^ against ALL reflections. The weighted *R*-factor *wR* and goodness of fit *S* are based on *F*^2^, conventional *R*-factors *R* are based on *F*, with *F* set to zero for negative *F*^2^. The threshold expression of *F*^2^ > σ(*F*^2^) is used only for calculating *R*-factors(gt) *etc*. and is not relevant to the choice of reflections for refinement. *R*-factors based on *F*^2^ are statistically about twice as large as those based on *F*, and *R*- factors based on ALL data will be even larger.

### Fractional atomic coordinates and isotropic or equivalent isotropic displacement parameters (Å^2^)

**Table:**

	*x*	*y*	*z*	*U*_iso_*/*U*_eq_	
Br1	0.41562 (5)	0.27735 (5)	0.15160 (5)	0.0940 (2)	
S1	1.07185 (8)	0.19371 (9)	0.35750 (8)	0.0644 (2)	
O1	−0.2430 (2)	0.1320 (2)	−0.12616 (19)	0.0664 (6)	
O2	−0.4997 (2)	0.1780 (2)	−0.22714 (18)	0.0617 (5)	
H1O2	−0.5807	0.1942	−0.2456	0.092*	
O3	1.0331 (3)	0.0475 (3)	0.3536 (3)	0.1036 (10)	
O4	1.1634 (3)	0.2910 (3)	0.4784 (2)	0.1020 (9)	
O5	1.1441 (3)	0.2289 (3)	0.2791 (3)	0.0922 (8)	
N1	0.3889 (2)	0.6912 (2)	0.43554 (19)	0.0449 (5)	
C1	0.4991 (3)	0.7901 (3)	0.5431 (2)	0.0542 (7)	
H1A	0.5957	0.7823	0.5812	0.065*	
C2	0.4719 (4)	0.8995 (3)	0.5961 (3)	0.0566 (7)	
H2A	0.5483	0.9660	0.6698	0.068*	
C3	0.3287 (4)	0.9101 (3)	0.5387 (3)	0.0586 (7)	
H3A	0.3080	0.9848	0.5732	0.070*	
C4	0.2177 (3)	0.8115 (3)	0.4318 (3)	0.0555 (7)	
H4A	0.1215	0.8199	0.3936	0.067*	
C5	0.2451 (3)	0.6974 (3)	0.3778 (2)	0.0455 (6)	
C6	0.1282 (3)	0.5869 (3)	0.2659 (2)	0.0478 (6)	
H6A	0.1532	0.5114	0.2388	0.057*	
C7	−0.0113 (3)	0.5867 (3)	0.2003 (2)	0.0492 (6)	
H7A	−0.0320	0.6648	0.2281	0.059*	
C8	−0.1367 (3)	0.4785 (3)	0.0895 (2)	0.0435 (6)	
C9	−0.1236 (3)	0.3541 (3)	0.0378 (2)	0.0443 (6)	
H9A	−0.0318	0.3384	0.0750	0.053*	
C10	−0.2452 (3)	0.2549 (3)	−0.0674 (2)	0.0446 (6)	
C11	−0.3846 (3)	0.2778 (3)	−0.1224 (2)	0.0446 (6)	
C12	−0.3978 (3)	0.3996 (3)	−0.0715 (2)	0.0484 (6)	
H12A	−0.4902	0.4148	−0.1079	0.058*	
C13	−0.2746 (3)	0.4999 (3)	0.0333 (2)	0.0513 (6)	
H13A	−0.2843	0.5825	0.0665	0.062*	
C14	0.4288 (3)	0.5777 (3)	0.3817 (3)	0.0575 (7)	
H14A	0.5370	0.5939	0.4304	0.086*	
H14B	0.4148	0.5782	0.3017	0.086*	
H14C	0.3607	0.4871	0.3780	0.086*	
C15	−0.1033 (4)	0.1044 (4)	−0.0758 (3)	0.0733 (10)	
H15A	−0.1162	0.0160	−0.1260	0.110*	
H15B	−0.0847	0.0984	0.0038	0.110*	
H15C	−0.0147	0.1802	−0.0712	0.110*	
C16	0.8901 (3)	0.2169 (3)	0.3001 (2)	0.0474 (6)	
C17	0.8818 (4)	0.3438 (3)	0.3475 (3)	0.0577 (7)	
H17A	0.9713	0.4164	0.4103	0.069*	
C18	0.7421 (4)	0.3636 (3)	0.3025 (3)	0.0649 (8)	
H18A	0.7365	0.4492	0.3339	0.078*	
C19	0.6110 (3)	0.2548 (3)	0.2102 (3)	0.0568 (7)	
C20	0.6163 (3)	0.1274 (3)	0.1621 (3)	0.0547 (7)	
H20A	0.5259	0.0546	0.1002	0.066*	
C21	0.7575 (3)	0.1089 (3)	0.2070 (2)	0.0519 (6)	
H21A	0.7633	0.0238	0.1744	0.062*	
O1W	0.2172 (3)	0.1988 (3)	0.6854 (3)	0.0753 (7)	
H2W1	0.196 (5)	0.217 (5)	0.622 (4)	0.093 (16)*	
H1W1	0.148 (5)	0.124 (4)	0.671 (3)	0.079 (13)*	

### Atomic displacement parameters (Å^2^)

**Table:**

	*U*^11^	*U*^22^	*U*^33^	*U*^12^	*U*^13^	*U*^23^
Br1	0.0699 (3)	0.1027 (3)	0.1419 (4)	0.0567 (2)	0.0504 (3)	0.0641 (3)
S1	0.0390 (4)	0.0670 (5)	0.0786 (6)	0.0186 (3)	0.0175 (4)	0.0237 (4)
O1	0.0524 (11)	0.0487 (11)	0.0694 (13)	0.0257 (9)	0.0008 (10)	−0.0031 (10)
O2	0.0436 (10)	0.0497 (11)	0.0594 (12)	0.0154 (9)	−0.0018 (9)	−0.0002 (9)
O3	0.0558 (14)	0.0819 (17)	0.171 (3)	0.0330 (13)	0.0319 (16)	0.0612 (19)
O4	0.0677 (16)	0.118 (2)	0.0798 (18)	0.0354 (16)	−0.0017 (14)	0.0105 (16)
O5	0.0644 (15)	0.112 (2)	0.122 (2)	0.0398 (15)	0.0553 (16)	0.0469 (18)
N1	0.0371 (11)	0.0485 (12)	0.0416 (12)	0.0127 (9)	0.0123 (9)	0.0139 (10)
C1	0.0380 (14)	0.0643 (18)	0.0439 (15)	0.0100 (13)	0.0079 (12)	0.0178 (13)
C2	0.0526 (16)	0.0527 (16)	0.0403 (15)	0.0047 (13)	0.0113 (13)	0.0048 (12)
C3	0.0587 (18)	0.0557 (17)	0.0496 (16)	0.0162 (14)	0.0207 (14)	0.0056 (13)
C4	0.0462 (15)	0.0586 (17)	0.0508 (16)	0.0210 (13)	0.0139 (13)	0.0065 (13)
C5	0.0386 (13)	0.0493 (15)	0.0412 (14)	0.0135 (11)	0.0133 (11)	0.0125 (11)
C6	0.0393 (13)	0.0471 (14)	0.0441 (14)	0.0154 (11)	0.0103 (11)	0.0042 (11)
C7	0.0467 (14)	0.0477 (15)	0.0440 (14)	0.0187 (12)	0.0133 (12)	0.0062 (12)
C8	0.0391 (13)	0.0471 (14)	0.0377 (13)	0.0163 (11)	0.0118 (11)	0.0092 (11)
C9	0.0344 (12)	0.0466 (14)	0.0454 (14)	0.0168 (11)	0.0097 (11)	0.0137 (11)
C10	0.0416 (13)	0.0389 (13)	0.0461 (14)	0.0150 (11)	0.0130 (11)	0.0107 (11)
C11	0.0362 (12)	0.0407 (13)	0.0424 (14)	0.0091 (10)	0.0075 (11)	0.0104 (11)
C12	0.0361 (13)	0.0537 (15)	0.0470 (15)	0.0202 (12)	0.0092 (11)	0.0110 (12)
C13	0.0462 (15)	0.0524 (15)	0.0478 (15)	0.0246 (12)	0.0124 (12)	0.0063 (12)
C14	0.0448 (15)	0.0613 (17)	0.0609 (18)	0.0240 (13)	0.0149 (13)	0.0182 (14)
C15	0.0608 (19)	0.0602 (19)	0.084 (2)	0.0365 (16)	0.0133 (17)	0.0043 (17)
C16	0.0414 (13)	0.0529 (15)	0.0481 (15)	0.0163 (12)	0.0204 (12)	0.0178 (12)
C17	0.0547 (17)	0.0511 (16)	0.0597 (18)	0.0130 (13)	0.0259 (15)	0.0107 (14)
C18	0.070 (2)	0.0483 (16)	0.089 (2)	0.0268 (15)	0.0453 (19)	0.0212 (16)
C19	0.0525 (16)	0.0658 (19)	0.0734 (19)	0.0317 (15)	0.0359 (15)	0.0370 (16)
C20	0.0464 (15)	0.0619 (17)	0.0501 (16)	0.0190 (13)	0.0174 (13)	0.0159 (13)
C21	0.0498 (15)	0.0536 (16)	0.0513 (16)	0.0227 (13)	0.0217 (13)	0.0098 (13)
O1W	0.0510 (14)	0.0705 (17)	0.0782 (19)	0.0190 (13)	0.0064 (12)	0.0174 (14)

### Geometric parameters (Å, º)

**Table:**

Br1—C19	1.892 (3)	C8—C9	1.398 (4)
S1—O3	1.433 (3)	C9—C10	1.375 (4)
S1—O5	1.438 (3)	C9—H9A	0.9300
S1—O4	1.438 (3)	C10—C11	1.404 (4)
S1—C16	1.773 (3)	C11—C12	1.373 (4)
O1—C10	1.363 (3)	C12—C13	1.382 (4)
O1—C15	1.425 (3)	C12—H12A	0.9300
O2—C11	1.356 (3)	C13—H13A	0.9300
O2—H1O2	0.8200	C14—H14A	0.9600
N1—C1	1.360 (3)	C14—H14B	0.9600
N1—C5	1.361 (3)	C14—H14C	0.9600
N1—C14	1.473 (4)	C15—H15A	0.9600
C1—C2	1.355 (4)	C15—H15B	0.9600
C1—H1A	0.9300	C15—H15C	0.9600
C2—C3	1.376 (4)	C16—C21	1.380 (4)
C2—H2A	0.9300	C16—C17	1.381 (4)
C3—C4	1.357 (4)	C17—C18	1.373 (4)
C3—H3A	0.9300	C17—H17A	0.9300
C4—C5	1.400 (4)	C18—C19	1.374 (5)
C4—H4A	0.9300	C18—H18A	0.9300
C5—C6	1.449 (4)	C19—C20	1.374 (4)
C6—C7	1.318 (4)	C20—C21	1.380 (4)
C6—H6A	0.9300	C20—H20A	0.9300
C7—C8	1.454 (4)	C21—H21A	0.9300
C7—H7A	0.9300	O1W—H2W1	0.81 (4)
C8—C13	1.385 (4)	O1W—H1W1	0.80 (4)
			
O3—S1—O5	112.54 (19)	O2—C11—C12	122.9 (2)
O3—S1—O4	112.9 (2)	O2—C11—C10	117.3 (2)
O5—S1—O4	112.02 (19)	C12—C11—C10	119.8 (2)
O3—S1—C16	106.57 (14)	C11—C12—C13	120.4 (2)
O5—S1—C16	105.90 (14)	C11—C12—H12A	119.8
O4—S1—C16	106.35 (15)	C13—C12—H12A	119.8
C10—O1—C15	117.7 (2)	C12—C13—C8	120.6 (2)
C11—O2—H1O2	109.5	C12—C13—H13A	119.7
C1—N1—C5	121.0 (2)	C8—C13—H13A	119.7
C1—N1—C14	118.6 (2)	N1—C14—H14A	109.5
C5—N1—C14	120.4 (2)	N1—C14—H14B	109.5
C2—C1—N1	121.7 (3)	H14A—C14—H14B	109.5
C2—C1—H1A	119.1	N1—C14—H14C	109.5
N1—C1—H1A	119.1	H14A—C14—H14C	109.5
C1—C2—C3	118.6 (3)	H14B—C14—H14C	109.5
C1—C2—H2A	120.7	O1—C15—H15A	109.5
C3—C2—H2A	120.7	O1—C15—H15B	109.5
C4—C3—C2	120.1 (3)	H15A—C15—H15B	109.5
C4—C3—H3A	120.0	O1—C15—H15C	109.5
C2—C3—H3A	120.0	H15A—C15—H15C	109.5
C3—C4—C5	121.3 (3)	H15B—C15—H15C	109.5
C3—C4—H4A	119.4	C21—C16—C17	120.0 (3)
C5—C4—H4A	119.4	C21—C16—S1	120.0 (2)
N1—C5—C4	117.3 (2)	C17—C16—S1	120.0 (2)
N1—C5—C6	119.4 (2)	C18—C17—C16	120.4 (3)
C4—C5—C6	123.3 (2)	C18—C17—H17A	119.8
C7—C6—C5	124.1 (3)	C16—C17—H17A	119.8
C7—C6—H6A	118.0	C17—C18—C19	118.9 (3)
C5—C6—H6A	118.0	C17—C18—H18A	120.6
C6—C7—C8	127.6 (3)	C19—C18—H18A	120.6
C6—C7—H7A	116.2	C18—C19—C20	121.7 (3)
C8—C7—H7A	116.2	C18—C19—Br1	119.4 (2)
C13—C8—C9	118.9 (2)	C20—C19—Br1	118.8 (2)
C13—C8—C7	118.4 (2)	C19—C20—C21	119.1 (3)
C9—C8—C7	122.7 (2)	C19—C20—H20A	120.5
C10—C9—C8	120.7 (2)	C21—C20—H20A	120.5
C10—C9—H9A	119.7	C16—C21—C20	119.9 (3)
C8—C9—H9A	119.7	C16—C21—H21A	120.0
O1—C10—C9	125.3 (2)	C20—C21—H21A	120.0
O1—C10—C11	115.1 (2)	H2W1—O1W—H1W1	105 (4)
C9—C10—C11	119.6 (2)		
			
C5—N1—C1—C2	−1.1 (4)	O1—C10—C11—C12	−178.8 (2)
C14—N1—C1—C2	178.2 (3)	C9—C10—C11—C12	0.8 (4)
N1—C1—C2—C3	−0.2 (4)	O2—C11—C12—C13	−177.9 (3)
C1—C2—C3—C4	0.6 (5)	C10—C11—C12—C13	0.1 (4)
C2—C3—C4—C5	0.2 (5)	C11—C12—C13—C8	−0.7 (4)
C1—N1—C5—C4	1.9 (4)	C9—C8—C13—C12	0.5 (4)
C14—N1—C5—C4	−177.4 (3)	C7—C8—C13—C12	−179.4 (3)
C1—N1—C5—C6	−177.4 (2)	O3—S1—C16—C21	−36.2 (3)
C14—N1—C5—C6	3.3 (4)	O5—S1—C16—C21	83.9 (3)
C3—C4—C5—N1	−1.5 (4)	O4—S1—C16—C21	−156.8 (2)
C3—C4—C5—C6	177.8 (3)	O3—S1—C16—C17	144.8 (3)
N1—C5—C6—C7	−176.7 (3)	O5—S1—C16—C17	−95.2 (3)
C4—C5—C6—C7	4.1 (4)	O4—S1—C16—C17	24.2 (3)
C5—C6—C7—C8	−178.0 (3)	C21—C16—C17—C18	0.0 (4)
C6—C7—C8—C13	−179.1 (3)	S1—C16—C17—C18	179.0 (2)
C6—C7—C8—C9	1.0 (5)	C16—C17—C18—C19	0.4 (5)
C13—C8—C9—C10	0.4 (4)	C17—C18—C19—C20	−0.1 (5)
C7—C8—C9—C10	−179.8 (2)	C17—C18—C19—Br1	177.5 (2)
C15—O1—C10—C9	−0.8 (4)	C18—C19—C20—C21	−0.6 (5)
C15—O1—C10—C11	178.8 (3)	Br1—C19—C20—C21	−178.2 (2)
C8—C9—C10—O1	178.6 (3)	C17—C16—C21—C20	−0.7 (4)
C8—C9—C10—C11	−1.0 (4)	S1—C16—C21—C20	−179.7 (2)
O1—C10—C11—O2	−0.8 (4)	C19—C20—C21—C16	1.0 (4)
C9—C10—C11—O2	178.8 (2)		

### Hydrogen-bond geometry (Å, º)

**Table:**

*D*—H···*A*	*D*—H	H···*A*	*D*···*A*	*D*—H···*A*
O2—H1*O*2···O1*W*^i^	0.82	1.88	2.685 (4)	169
O1*W*—H2*W*1···O4^ii^	0.81 (5)	2.03 (5)	2.834 (4)	172 (5)
O1*W*—H1*W*1···O3^iii^	0.81 (4)	1.99 (5)	2.793 (5)	173 (5)
C1—H1*A*···O5^iv^	0.93	2.57	3.491 (4)	170
C2—H2*A*···O1^v^	0.93	2.51	3.440 (4)	176
C2—H2*A*···O2^v^	0.93	2.60	3.183 (4)	121
C3—H3*A*···O2^v^	0.93	2.54	3.160 (4)	124
C14—H14*A*···O4^iv^	0.96	2.54	3.448 (5)	158

Symmetry codes: (i) *x*−1, *y*, *z*−1; (ii) *x*−1, *y*, *z*; (iii) −*x*+1, −*y*, −*z*+1; (iv) −*x*+2, −*y*+1, −*z*+1; (v) *x*+1, *y*+1, *z*+1.
